# Supplementary material for: Novel Alumina Matrix Composites Reinforced with MAX Phases—Microstructure Analysis and Mechanical Properties
Source: Materials (Basel). 2022 Oct 5;15(19):6909. doi: 10.3390/ma15196909 (PMC9571909; doi:10.3390/ma15196909)
Supplement: Supplementary file 1 [file materials-15-06909-s001.zip › materials-1935958-supplementary.pdf]

materials

MDPI

Supplementary Material

Novel Alumina Matrix Composites Reinforced with MAX Phases—Microstructure Analysis and Mechanical Properties

Mateusz Petrus [1], Jaroslaw Wozniak [1,*], Tomasz Cygan [1], Wojciech Pawlak [2] and Andrzej Olszyna [1]

[1] Faculty of Material Science and Engineering, Warsaw University of Technology, ul. Wołoska 141, 02-507 Warsaw, Poland
[2] Faculty of Mechanical Engineering, Lodz University of Technology, Stefanowskiego 1/15 St, 90-924 Lodz, Poland
* Correspondence: jaroslaw.wozniak@pw.edu.pl

Citation: Petrus, M.; Wozniak, J.; Cygan, T.; Pawlak, W.; Olszyna, A. Novel Alumina Matrix Composites Reinforced with MAX Phases—Microstructure Analysis and Mechanical Properties. *Materials* **2022**, *15*, 6909. https://doi.org/10.3390/ma15196909

Academic Editor: Yin Fan

Received: 09 September 2022
Accepted: 30 September 2022
Published: 5 October 2022

Publisher's Note: MDPI stays neutral with regard to jurisdictional claims in published maps and institutional affiliations.

Copyright: © 2022 by the authors. Licensee MDPI, Basel, Switzerland. This article is an open access article distributed under the terms and conditions of the Creative Commons Attribution (CC BY) license (https://creativecommons.org/licenses/by/4.0/).

Supplementary information

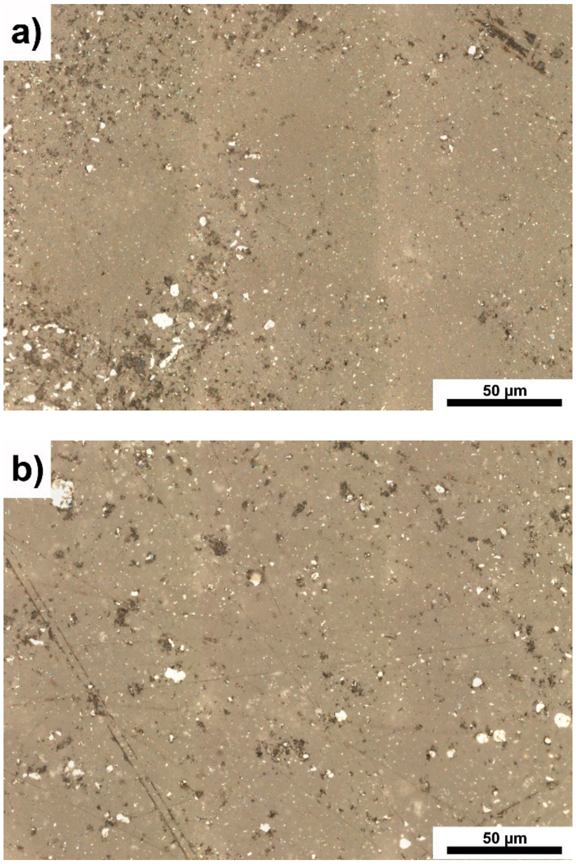

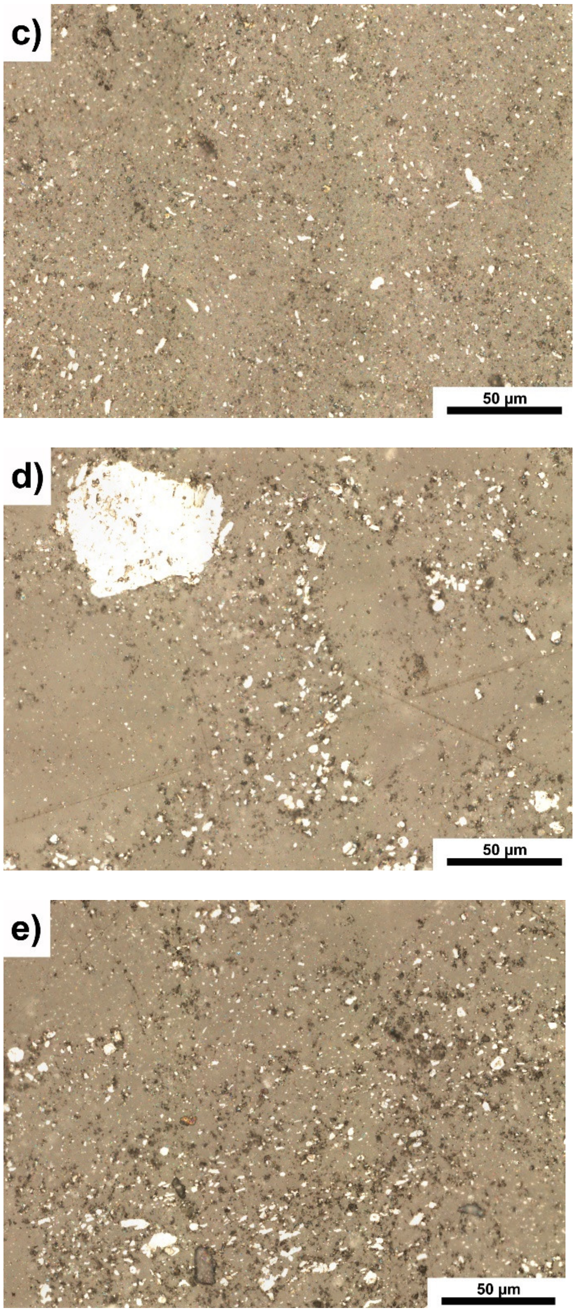
c)
50 µm
d)
50 µm
e)
50 µm

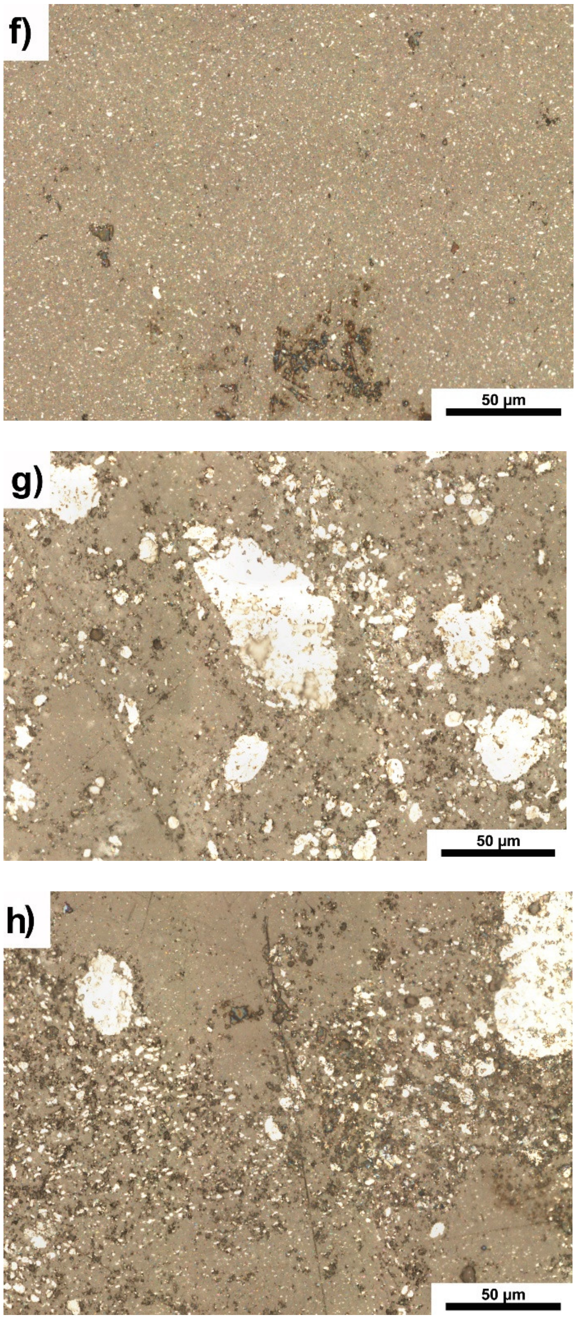
f)
50 µm
g)
50 µm
h)
50 µm

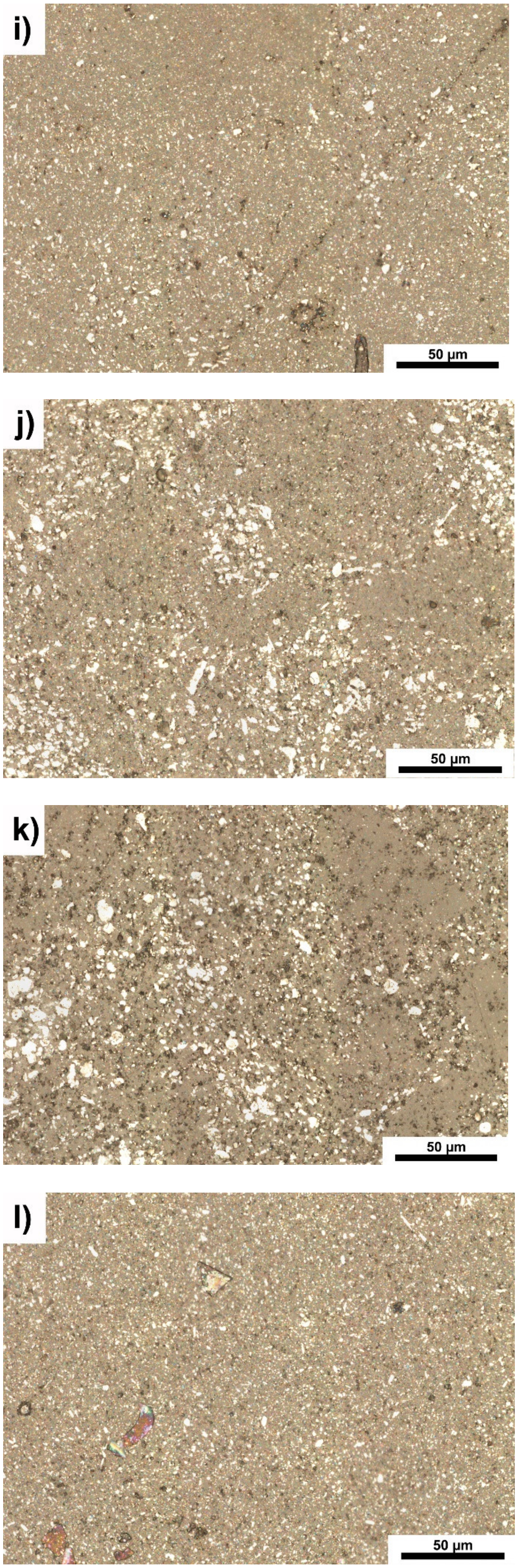
i)
50 µm
j)
50 µm
k)
50 µm
l)
50 µm

Scheme S1. AlC_2 phase for obtained composites. **a**) Al_2O_3 — 5wt% Ti_3AlC_2 from attritor mill; **b**) Al_2O_3 — 5wt% Ti_3AlC_2 from horizontal mill; **c**) Al_2O_3 — 5wt% Ti_3AlC_2 from planetary mill; **d**)Al_2O_3 — 10wt% Ti_3AlC_2 from attritor mill; **e**) Al_2O_3 — 10wt% Ti_3AlC_2 from horizontal mill; **f**) Al_2O_3 — 10wt% Ti_3AlC_2 from planetary mill; **g**) Al_2O_3 — 15wt% Ti_3AlC_2 from attritor mill; **h**) Al_2O_3 — 15wt% Ti_3AlC_2 from horizontal mill; **i**) Al_2O_3 — 15wt% Ti_3AlC_2 from planetary mill; **j**) Al_2O_3 — 20wt% Ti_3AlC_2 from attritor mill; **k**) Al_2O_3 — 20wt% Ti_3AlC_2 from horizontal mill; **l**) Al_2O_3 — 20wt% Ti_3AlC_2 from planetary mill
